# Drug Utilization Studies in Pregnant Women for Newly Licensed Medicinal Products: A Contribution from IMI ConcePTION

**DOI:** 10.1155/2024/8862801

**Published:** 2024-01-11

**Authors:** Sandra Lopez-Leon, Anja Geldhof, Julie Scotto, Keele Wurst, Meritxell Sabidó, Jingping Mo, Ditte Molgaard-Nielsen, Jorieke E. H. Bergman, Xuan Anh Phi, Sue Jordan

**Affiliations:** ^1^Novartis Pharmaceuticals, East Hanover, NJ, USA; ^2^Rutgers Center for Pharmacoepidemiology and Treatment Science, Rutgers University, New Brunswick, NJ, USA; ^3^Janssen Biologics B.V., Leiden, Netherlands; ^4^Bristol Myers Squibb, Princeton, NJ, USA; ^5^GlaxoSmithKline, Research Triangle Park, North Carolina, USA; ^6^Merck Healthcare KGaA, Darmstadt, Germany; ^7^Pfizer Inc., New York, NY, USA; ^8^Novo Nordisk, Søborg, Denmark; ^9^Department of Genetics, University of Groningen, University Medical Center Groningen, Groningen, Netherlands; ^10^Faculty of Medicine, Health and Life Sciences, Swansea University, Swansea, Wales, UK

## Abstract

**Purpose:**

Studies focusing on safety outcomes typically require large populations to comprehensively characterise the patient groups exposed to the medicines under investigation. However, there is often less information for subpopulations, such as pregnant or breastfeeding women, particularly when new medicines are considered. It is important to understand what information can be obtained from drug utilization studies (DUS) involving pregnant women in the early years postmarketing to provide supportive information for safety studies. The aims of this literature review are to (1) identify and review DUS for new medicines in pregnancy and breastfeeding and (2) list and summarise key information items to be reported in a DUS for new medicines in pregnancy.

**Methods:**

To identify postmarketing DUS of new prescription medicines or enantiomers in pregnancy, a systematic literature review was undertaken in PubMed and Embase between January 2015 and June 2022. In addition, the complete database of the ENCePP EU PAS Register was systematically searched to June 2022.

**Results:**

We identified 11 published DUS on new medicines in pregnancy from the ENCePP EU PAS Register and none from other sources. No studies on breastfeeding were identified. The 11 identified publications reported the medicine's use for the first 3 to 5 years after marketing approval. No reports assessed utilization in the first 3 years of approval. It was usual to issue interim reports annually (7 studies). All studies concerned conditions managed in ambulatory care (primary care and outpatient facilities) and included some primary care prescribing. Most (*n* = 8) only had prescribing/dispensing data available at individual level for ambulatory care; outpatient prescribing was included in three of these studies Three studies held a limited amount of in-hospital prescribing data. A DUS can confirm at an early stage whether there are sufficient exposed pregnancies in available data sources to ensure a safety study is powered to detect a difference in the prevalence of adverse pregnancy or infant outcomes or if additional data from other databases are needed. A DUS may also help address methodological considerations such as selection of comparators. DUS can be performed embedded in a DUS in the general population, in a cohort of women of childbearing age, or in a cohort of pregnant women.

**Conclusion:**

This review summarises key aspects of a DUS for new medicines in pregnancy. DUS for new medicines in pregnancy should be planned before marketing, scheduled for the first 3 to 5 years after release, with annual interim/progress reports, and reported in peer-reviewed journals. By offering detailed information on data sources, exposure timing, prevalence and location, coprescribing, comorbidities, coexposures, and demographics, a DUS will offer a firm foundation for safety studies and will help to contextualize spontaneous reporting of serious adverse events.

## 1. Introduction

Humans are exposed to new medicines for the first time in the context of preapproval clinical trials. However, some 95% of the clinical trials exclude pregnant and breastfeeding women due to ethical, legal, and safety concerns of reproductive toxicity of the new medicine [1]. Therefore, information regarding the use of new medicines during pregnancy and breastfeeding is often listed as missing in the summary of product characteristics, and a postapproval plan is needed to evaluate the safety of the newly approved product.

During drug development and before marketing authorization is granted, the safety of a new medicine is evaluated in preclinical studies, including reproductive toxicity studies in mammals. In addition, the safety information for new medicines may be supplemented by pooling data from patients who unexpectedly become pregnant during the preapproval clinical trials [2]. In the absence of safety concerns or signals related to pregnancy exposure during drug development, once the new medicine is on the market, it is subjected to safety monitoring using pharmacovigilance procedures [3]. Solicited or spontaneous reports of suspected adverse drug reactions (ADRs), including adverse pregnancy outcomes (e.g., congenital anomalies) and pregnancy exposure with and without ADRs, are collected by marketing authorization holders and regulatory authorities (such as the EMA); however, it is estimated that only 5% of all ADRs are spontaneously reported [4], and reporting is vulnerable to bias [5]. Therefore, there is a need to supplement spontaneous reports and solicited reports of ADRs, with data from other sources [6], for example, by secondary use of routinely collected healthcare data, to further investigate the potential harms, risks, and benefits. Use of whole-population data removes selection bias including volunteer bias [7]. Even if use of a medicine is not recommended or contraindicated in pregnancy, women unaware of their pregnancies may receive a prescription or there may be no alternative to treat maternal conditions. Therefore, many new medicines are first used in secondary or tertiary care, under specialist supervision; however, some healthcare databases do hold information on hospital prescribing. DUS were defined by the World Health Organization as studies which study the marketing, distribution, prescription, and use of medicinal products in a society, with special emphasis on the resulting medical and socioeconomic consequences.

The thalidomide tragedy may have faded from public consciousness, but the failure to act on the teratogenicity and neurodevelopmental harms of valproic acid derivatives for over two decades (from 1984 to 2018) indicates that the problem persists [8]. It has been reported that it takes on average 27 years (95% confidence interval (CI) 26-28 years) to determine the safety of a new medicine in pregnant women [9]; therefore, there is a need to help expedite the safety evaluation for new medicines and enantiomers. A drug utilization study (DUS) can help by adding efficiency to medication safety evaluation during pregnancy.

Several guidelines [10–12] and books [13, 14] consider DUS in the general, nonpregnant population. The book *Drug Utilization Research: Methods and Applications* presents a broad introduction to drug utilization measures and research [14]. The World Health Organization's guideline focuses on establishing a DUS [10]. ENCEPP SafeGUARD provides examples of DUS [12], and Rasmussen et al. list the core concepts that can be used in DUS; these authors list appropriate analytical approaches for designing a DUS (e.g., basic epidemiological measures, adherence, combinations of medicines, switching, polypharmacy, and drug misuse) [11]. A tool was developed in 2000 to assess the quality of DUS and drug utilization reviews; however, this tool was created for primary data collection, often in relation to audit or prescribing quality, rather than pharmacoepidemiological studies involving routine healthcare databases [15]. The EQUATOR network offers guidelines on observational studies [16] and pharmacoepidemiological studies using routine healthcare data [17]. To our knowledge, there is no specific guideline for DUS during pregnancy using secondary data sources that details items to be captured, recorded, and reported. The objectives of this literature review are to (1) identify and review DUS for new medicines in pregnancy and breastfeeding, (2) list and summarise key information items to be reported in a DUS for new medicines (in the last 5 years) in pregnancy, and (3) report on the earliest timeframe between introduction to the market and evaluation of DUS during pregnancy.

This publication is part of the Innovative Medicines Initiative (IMI) ConcePTION project, which aims to build an ecosystem for better monitoring and communication of medication safety in pregnancy and breastfeeding. The results of this study will help guide future DUS for new drugs in pregnancy and potentially breastfeeding (breastfeeding was omitted due to lack of data).

## 2. Methods

### 2.1. Literature Search

To identify postmarketing DUS of new medicines in pregnancy, a systematic review was undertaken in two stages. In 2022, PubMed, Embase, and the ENCePP EU PAS Register were searched. The publication date was limited to 01.01.2015 through 01.07.2022. The search was restricted to publications in English (for further details on the search strategy, see Appendix). The inclusion criterion was assessment of the utilization of new medicines during pregnancy or breastfeeding. “New” medicines were defined as those that reached the market within 5 years of the date of data analysis or review. New medicines include new molecules, enantiomers, generics/biosimilars, and formulations. In addition, data were collected on routes of administration and indications. In the ENCePP EU PAS Register [18], “pregnancy” were selected as “other population” and “drug utilization study” was selected under “scope of study.” (The register did not have an option to search “breastfeeding/lactation”.) Studies identified via the ENCePP EU PAS Register were included if they mentioned that they were going to study drug utilization in pregnancy or breastfeeding in their protocol.

### 2.2. Screening

Studies with only the abstract available were excluded.

All titles and abstracts were initially screened to identify DUS. Full texts of potentially relevant studies were then screened to determine final eligibility. Two reviewers (SLL, JS) independently reviewed the search results for inclusion narrowing potential studies successively in three stages: by title, by abstract, and by full manuscript.

### 2.3. Data Extraction

Five reviewers (JS, KW, MS, JM, and DM) undertook data extraction from the included studies using a data extraction Excel sheet. Information on key aspects of a DUS was extracted from the title, introduction, methods, tables, results, and discussion of each included study. The key aspects of the studies were the pertinent items of information reported. Each study was scrutinized by two researchers independently, and findings were compared. The findings were consistent, and there were no disagreements.

### 2.4. Data Synthesis

All the extracted information from the included studies was captured in Excel tables.

### 2.5. Developing Recommendations

Information items reported in the published studies and protocols were tabulated in an Excel file. Once the items were identified, we returned to all 11 studies and calculated the number of studies reporting each information item. This number is included next to each item and helps us understand which items are commonly included.

## 3. Results

**Figure 1:**
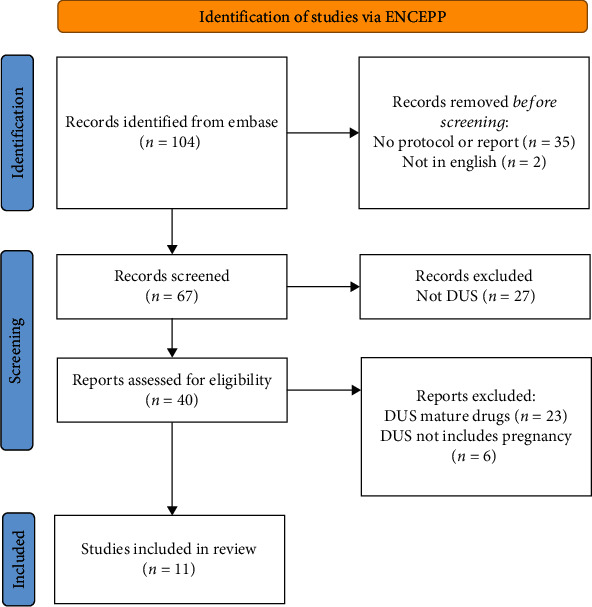
Selection of studies: flow diagram.

After removing the duplicates, a total of 2,284 unique potentially relevant studies were identified in PubMed and Embase. However, none of the studies were DUS for new drugs. The search in the ENCePP EU PAS Register identified 104 studies. Thirty-seven studies were excluded because they did not include a study concept, protocol, or study report (*n* = 35) or because they were not in English (*n* = 2). Of the 67 studies screened, 40 were DUS. Twenty-three were excluded because they did not study a new drug, and 6 were excluded because they did not included data on either pregnancy or breastfeeding (Figure 1, PRISMA).

In total, 11 DUS posted in the ENCEPP EU PAS Register within 3 years after EMA medicine approval were included for review. There were 3 studies including information on use of medications during pregnancy in which only the protocol was available; for these, we extracted information related to the methods. The other 8 studies presented study reports. For all these, information was extracted from these to describe the components to be considered when conducting DUS of new medicines. No studies considering breastfeeding were identified.

The patients were prescribed either a specific medicine or a drug class. There was only one study that exclusively studied pregnant women (study 27574, Table 1) and one study that focused on women of childbearing age (study 11841, Table 1). The other studies evaluated drug utilization in the general population for all users of the medicine(s) in question and included a subgroup analysis of the estimated prevalence of prescribing the medicine of interest to pregnant women. Four studies reported that there were no pregnant women prescribed the medicine (studies 4845, 13783, 9507, and 17062, Table 1), and two did not mention pregnant women in the report but did in their protocol (studies 12839 and 14445, Table 1). One study identified 462 pregnant women (study 4270, Table 1), and one identified 1 pregnant woman (study 3901, Table 1). The studies identifying zero pregnancies within the first 3-4 years of marketing were for the drugs fidaxomicin, dulaglutide, and glycopyrronium bromide.

The studies identified addressed conditions largely managed in ambulatory care (primary care and/or outpatient facilities), mainly long-term conditions: migraine (2-36014, 27574), type 2 diabetes (3-13783, 14445, and 11841), thrombosis (1-17062), chronic obstructive pulmonary disease/COPD (1-4845), irritable bowel syndrome/IBS (1-12839), cystic fibrosis (1-4270), gastric acidity (1-3901), and *Clostridium difficile* (1-9507). All studies included some primary care prescribing. Most studies (*n* = 8) only had prescribing/dispensing data available at individual level for ambulatory care; three of these included some outpatient prescribing (4845, 27574, and 12839). Three studies held a limited amount of in-hospital prescribing data (17062, 4270), sometimes for just 1 participating centre (36014). Primary care studies included whole populations and in several countries. The duration of the studies ranged from 3 to 5 years and most reported interim results annually. No reports addressed utilization of new medicines during breastfeeding. Details of these 11 studies can be found in Table 1, appended.

### 3.1. Information Included in DUS for New Medicines in Pregnancy

Items of information useful to or needed in a DUS for a medicine new to market identified in at least one study are presented below, in the order of the sections of a pharmacoepidemiological study protocol. Since not all DUS have the same objectives, there can be no “one size fits all,” and studies should be evaluated case by case. The column on the right, in all the tables below, represents the total number of studies presenting that item, from a total of 11.

#### 3.1.1. Items for a DUS: Title and Abstract

We recommend that study type be specified with commonly used terms, such as “Drug Utilization Study”, “observational”, and “pregnancy”. The abstract should name the specific medicine(s)/class, indication(s), geographical region(s), setting, and databases deployed, as in all the studies identified.

#### 3.1.2. Items for a DUS: Introduction

While describing the background and rationale for the DUS, the items in Table 2 would be helpful, particularly both information on nonclinical and clinical safety data on the medicine and similar medicines during pregnancy. If information on safety in pregnancy or breastfeeding is unknown, this should be stated.

For a new medicine on the market, readers will need to understand when the new medicine was approved by regulatory authorities and when it was launched and available in the countries/regions under investigation. There are cases where there is a considerable delay between the regulatory approval date and the availability of the new medicine in routine care, mainly driven by reimbursement decisions [19], guidelines, or prescribers' caution. There may be further delays before data are available for research, due to time lag in the databases.  Table 2 lists information that is pertinent to contextualization of the results of a DUS. If information is unobtainable or unknown, this should be noted.

The last part of the introduction should explain the purpose of conducting the DUS.  Table 3 describes appropriate objectives for DUS. For example, a DUS can help determine whether the contributing databases are adequate for possible further safety studies on a new medicine (e.g., if the medicine and timing of administration are fully recorded).

#### 3.1.3. Items for a DUS: Methods and Results

*(1) Databases*.  Table 4 lists items to be considered in the methods and results section of a DUS for a new medicine. DUS typically use large population-based databases, e.g., prescription databases, reimbursement claims databases, or linked health service records. If the DUS is used to plan a future pregnancy safety study, the numbers of exposed and unexposed pregnancies in the DUS can be used to determine the achievable level of precision for the risk of predefined adverse pregnancy outcomes. If several databases are included, results should be stratified by country/database to identify similarities and differences between databases. This will determine if it is appropriate to plan a meta-analysis or aggregated multilevel analysis in further studies. Potential confounding factors should be included to address threats to validity: in real-world settings, any associations between maternal medicines and infant outcomes may be confounded by concomitant exposures and demographic factors. All possible confounding variables should be described by group, with or without inferential analyses.

#### 3.1.4. Items for a DUS: Considerations for Study Discussion

Table 5 describes topics that should be considered for inclusion in the discussion section.

## 4. Discussion

We identified very few published DUS designed to determine the use of new medicines in pregnant or breastfeeding women. Most studies were aimed at determining if pregnant women were taking the medicine of interest. From the eight studies that presented results, five did not identify any women prescribed the medicine of interest and two did not present any information on pregnancy in their study reports. Publication bias is possible given that studies identifying zero or very few exposed pregnant women may not be published or if <5 pregnant women exposed this may not be published due to governance restrictions on reporting of low numbers [20]. Low numbers of recorded exposures during pregnancy or breastfeeding during the medicine's first years on the market militates against safety assessments should the medicine be used by these vulnerable groups. Establishing consortia of databases including primary, secondary and tertiary care data for DUS in pregnancy and breastfeeding would address this problem [21, 22]. If no exposed pregnancies are identified across several whole-population databases, with primary, secondary, and tertiary care prescribing, it would suggest that the medicine is not being prescribed to pregnant women and further safety studies needing large samples would be premature; meanwhile, voluntary adverse event reporting databases should be scrutinized for serious adverse events, including congenital anomalies.

The studies identified addressed conditions generally managed in ambulatory care, and ten of 11 reported on long-term conditions. However, many new medicines are introduced in secondary and tertiary care, as this is where most severe conditions and sickest patients are treated. New medicines are most frequently developed for the most serious conditions: for example, in the UK, in 2021, 56 of 155 new products launched were to treat immunological and malignant disorders and 13 for the next highest categories (cardiovascular and infectious diseases) [23]. However, eight of 11 publications identified had no in-hospital prescribing data, and five had only primary care prescribing, although some of the regimens recorded may be initiated by specialists based in secondary or tertiary care (Table 1). DUS on new medicines for immunological and malignant disorders were not located, despite this being the largest prescribing category [23]. Large-scale patient data on hospital prescribing are scarce [24, 25]. Many routine healthcare databases, particularly in Europe, do not include in-hospital prescribing, and therefore, if the medicine is restricted to hospital use, it is not possible to undertake a DUS [26]. For example, most intravenous medicines, particularly anticancer treatments, are administered in hospitals and not reliably recorded in primary care. Therefore, restricting a DUS to ambulatory or primary care without first ascertaining use in secondary care may generate misleading and invalid conclusions regarding prevalence of exposure in pregnancy.

There was only one study that exclusively studied pregnant women and only one study that focused on women of childbearing age. The other nine studies evaluated drug utilization in the general population for all users of the medicine(s) in question: as a stratification they estimated the frequency of pregnant women prescribed the medicine of interest. Three of these studies stated in their protocol that they intended to assess utilization in pregnancy; however, when the results were published, no data on pregnancy was presented. EMA guidelines [27] on registry-based studies state that feasibility analyses may be submitted separately or as part of a proposed protocol for a registry-based study. However, it is also important to publish information derived from pregnancies in feasibility studies, as this can guide the protocols of future studies.

The methods used for DUS of older products are very similar to those identified for new products. Most DUS for new and established medicines focus on estimating the number of pregnant women prescribed the medicine. When new medicines are considered, initially few people are exposed, militating against a comprehensive evaluation of some areas of concern, particularly pregnancy and breastfeeding. The objective of a DUS for a new product should be to expedite, facilitate, and guide the assessment of safety concerns during pregnancy. Drug safety studies can be planned in parallel or sequentially using the same databases as the DUS. As observed in several of the studies, the protocol of a DUS can be written before the medicine is on the market. When planned as a phased approach, the main objective may be to determine when there is enough power to perform a safety study. Determining sample size ensures that risk estimates are precise: if a study is too small, it will yield wide confidence intervals and imprecise estimates. Other DUS objectives include identification and prevalence of risk factors that may be confounders such as comedications, comorbidities, smoking, age, education, body weight, substance misuse, and any differences between exposed and nonexposed populations (particularly women of childbearing age). The DUS will also generate knowledge of the unique characteristics of the data sources regarding medicine exposure and can establish the strengths and limitations of each database, so that a range of databases can be considered to account for known risk factors. For example, the Nordic databases hold data on paternity, while Wales holds data on substance misuse [28]. When a DUS is conducted in parallel with a safety study, the information can inform decision points as to phasing and interpreting the safety study results. However, complete and accurate data linkage is essential for safety studies [28].

### 4.1. Limitations and Strengths

The biggest limitation of this systematic review is that very few DUS for new drugs are being published. There were insufficient studies to illustrate good practice; however, the available studies offered an overview of the optimal structure for a DUS in this field (Tables 2–5) [16, 17]. These were considered in association with existing reporting guidelines [16, 17]. A central problem with DUS for new medicines in pregnancy is the absence of whole population databases for hospital prescribing. Prescription and administration data have been captured electronically for many years in most hospitals; however, even some well-established national healthcare databases have been unable to link this key data. Therefore, pharmacoepidemiologists can only investigate potential adverse effects of medicines amongst the less-ill primary care population, while key signals for harm are missed due to the fragmentation of data capture for the vulnerable, unwell hospital population. New technical, financial, and governance barriers are not immutable, and integrated databases may well emerge in the future. While the majority of pregnant women are healthy, a few experience serious health problems, whose treatment can only be evaluated from hospital prescribing data; the rarity of serious illness in pregnancy necessitates combining data from large populations. Comprehensive information is even more important for safety studies, particularly where prescribing is expected to be low or minimized due to manufacturers' warnings regarding unknown safety in pregnancy or contraindications. By accounting for all the items listed above, researchers increase the likelihood that the only remaining plausible explanation for harm identified is the prescribed medicine. We made no recommendation in relation to breastfeeding, because no studies reported drug utilization during or preceding breastfeeding: very few databases hold data on breastfeeding [29].

## 5. Conclusions

This review of DUS for medicines new to the market illustrates how DUS might add efficiency to the evaluation of medicines during pregnancy. A DUS supports safety studies by providing

- early confirmation as to whether there are sufficient exposed pregnancies to provide adequate statistical power to detect an association with an adverse pregnancy outcome
- context and information to evaluate whether additional data from other data sources or more time are needed
- information on the most appropriate data sources to use
- guidance on the optimal methods for a safety study, such as selection of comparator medicines or participants

These considerations are particularly relevant where adverse outcomes are relatively common, and with multiple aetiologies, for example, small for gestational age or absence of breastfeeding following antidepressant exposure [30]. This does not detract from the reporting of major congenital anomalies.

This paper offers suggestions as to considerations when planning and reporting DUS for new drugs in pregnant women. Such studies may be planned before marketing, either embedded in a DUS in the general population or in a retrospective cohort of pregnant women, typically for the first 3 to 5 years after release, with annual interim/progress reports. These reports may provide patient numbers or status progress updates to guide safety studies and augment interpretation of spontaneous pharmacovigilance reports, which start as soon as the drug is marketed. The results of these interim reports/final reports should be available in peer reviewed journals, so that researchers and physicians can access the information easily. DUS for new drugs and safety studies can be planned with a phased or parallel approach to expedite the knowledge generation regarding the safety of medicines used during pregnancy and breastfeeding.

## Figures and Tables

**Table 1:** Drug utilization studies for new drugs identified.

EU PAS number, year, documents	EMA approval of drug	Study objective(s)	Populations	Country/region	Database	Medicine studied, indication	Primary care/hospital prescribing	Duration (years)	Interim reports	Comparator groups	Number of pregnant women in report
36014, 2020, protocol	2018	Exploratory: frequency of pregnancies, estimate rates of pregnancy outcomes	Patients prescribed drug class	Denmark, Sweden, Finland, Norway	Danish, Swedish, Finnish, and Norwegian national healthcare databases	Erenumab, migraine	Primary, secondary, inpatient, outpatient Hospital treatment identified only in Denmark	4	Yearly after 2 years	Cohorts with same disease other treatments	Protocol

27574, 2020, protocol	2018	Primary objective: exposure to medicine during pregnancy	Pregnant patients with disease	US (submitted to EMA)	HealthCore Integrated Research Database (HIRD)	Galcanezumab, migraine	Primary care: outpatient prescription drug dispensing	5	Yearly	No	Protocol

17062, 2017, interim report	2015	Primary objective: characterise users (including pregnancy)	Patients prescribed the medicine	Belgium, Germany, Italy, Portugal, Spain, Switzerland, UK	Retrospective chart reviews	Edoxaban, anticoagulant	Hospitals and general practitioners, internal medicine, and specialists	3	After 1 year	No	0

9507, 2015 report	2012	Secondary objective: exposure to medicine during pregnancy	Patients prescribed the medicine	Spain, Germany, UK, Austria	Retrospective chart review	Fidaxomicin, Clostridium difficile	Primary care	4	No	No	0

13783, 2016, protocol and report	2014	Primary: frequency of use in different groups of patients (including pregnancy)	Patients prescribed the medicine	France, Germany, Spain, UK, Sweden	IMS Disease Analyzer, SIDIAP, NPR, SPDR, CPRD	Dulaglutide, T2DM	Primary care	3	Yearly	No	0

12839, 2016, protocol and report	2013	Primary: frequency of use in different groups of patients (including pregnancy)	Patients prescribed the medicine	UK, Spain, and Sweden	SIDIAP, CPRD, Sweden (NPR, PDR)	Linaclotide, irritable bowel syndrome	Primary care in UK and Spain, prescribed medicines in primary health care centers and outpatient specialists in Sweden	5	No	No	NR not mentioned in results

14445, 2016, protocol and report	2014	Secondary: frequency of use in different groups of patients (including pregnancy)	Patients prescribed the medicine	Germany, the Netherlands, Italy, England (UK)	BIPS, PHARMO, LHU, M-PEM	Dulaglutide, T2DM	Healthcare and administrative databases, for UK, primary care	NR	No	No	NR not mentioned in results

11841, 2015, protocol	2014	Primary: frequency of pregnancies, estimate rates of pregnancy outcomes	Patients with disease of childbearing age	UK	CPRD	Albiglutide, T2M	Primary care	4	Every 6 months	No	Protocol

4270, 2013, protocol and report	2012	Secondary objective frequency of pregnancy	Patients prescribed the medicine	US, Ireland, France, UK	ECFSPR Registry	Ivacaftor, cystic fibrosis	Specialists, inpatient and outpatient data	5	Yearly	Patients with disease not prescribed the medicine	462

4845, 2013, protocol and report	2012	Secondary objective frequency of pregnancy	Patients prescribed the medicine	UK, Denmark, Italy, the Netherlands, Spain	THIN, HSD, IPCI, Aarhus, SIDIAP	Glycopyrronium bromide, COPD	Outpatient (primary care) prescribing (specialists incomplete)	4	Yearly	No	0

3901, 2013, protocol and report	2012	Primary: frequency of pregnancy	Patients prescribed the medicines	France	SNIIR-AM	Bismuth subcitrate potassium, Helicobacter pylori	Nonhospital claims	3	Yearly	No	1

BIPS: Leibniz-Institute for Prevention Research and Epidemiology Germany; CPRD: Clinical Practice Research Datalink; ECFSPR: European Cystic Fibrosis Foundation Patient Registry; LHU: Caserta Local Health Unit Italy; M-PEM: Modified Prescription Event Monitoring Study; NPR: National Patient Register; NR: not reported; PDR: Prescription Drug Registry; SIDIAP: System for Development of Research in Primary Care; SPDR: Swedish Prescribed Drug Register; SNIIR-AM: Système National d'Information Inter-Régimes de l'Assurance Maladie; T2DM: type 2 diabetes mellitus; THIN: The Health Improvement Network.

**Table 2:** Items for the introduction.

	Items of information	Number of reports (*n* = 11)
The new medicine(s)	Approved indication(s) with approval and launch dates	9
	Date medicine first appears in the country/region/reimbursement database(s)	6
	Other treatment(s) for same indication(s)	7
	Regulatory requirements for the approval of medicine, e.g., postauthorization requirements	11
	Risk minimization measures, e.g., advice to “avoid” or “use only if benefits outweigh risk,” as recommended by the manufacturers or regulators	4
	Pharmacokinetic parameters particularly placental transfer, the elimination half-life, and volume of distribution for each trimester	3
	Safety information related to pregnancy from preapproval animal or clinical studies	4
	Data on adverse drug reactions (ADRs), particularly those related to pregnancy or breastfeeding from spontaneous reporting systems, such as the EudraVigilance database, and pregnancy registries for the medicine in question, medicines of the same class or for the same indication	2
	Summary of existing phase 3 trial data, both benefits and risks (ADRs) for women of childbearing age. If not available, for the whole population	2

Prevalence of the condition for which the medicine is approved or used for	In women of childbearing age and in pregnant women (before, during, and after pregnancy)	2
	Specify the denominator used, as applicable: general population, women in the general population, women in reproductive age, or women prescribed the medicine of interest	4
	In the country where the DUS is conducted (women of childbearing age and/or pregnant women)	6
	International variation	4

Prevalence of drug use	In women of childbearing age	2
	All women	1
	In pregnant women (before, during, and after pregnancy)	3
	International variation	1

**Table 3:** Possible objectives of a drug utilization study in pregnancy.

To assess if pregnant women are being prescribed or dispensed a specific new medicine	8
To assess the feasibility of conducting a pregnancy safety study for a newly approved medicine (are pregnant women and/or women of childbearing age using the medicine) by generating data for a sample size calculation	2
To estimate the prevalence of use amongst pregnant women or all women or those of childbearing age	8
To describe patterns of drug use in childbearing/pregnant women (dose, formulation, routes of administration, repeat prescriptions, trimester of use, and switching to other medicines or discontinuing any or all medicines)	4
To estimate the prevalence of the condition amongst women of childbearing age, and the proportion using the medicine, those of a similar class, and other medicines for the condition	3
To estimate the prevalence of drug use (or drug class) in women before, during (first, second, and third trimester), and/or after pregnancy and during breastfeeding	5
To estimate the prevalence of drug (or drug class) use in women of childbearing age or pregnant women by subgroups, e.g., socioeconomic status, ethnicity, clinical conditions, indications, concomitant prescribing, smoking or substance misuse, demographics including age, country/region, rurality, calendar years	2
To assess potential comparator groups for safety studies in pregnancy when it is not clear which group of patients would be the best comparators (e.g., patients with the disease not taking a medicine, or using other medicines)	3
To describe and/or compare drug utilization in populations, over time and/or according to other characteristics such as socioeconomic status	4
To compare the patient profiles of pregnant women using the medicine of interest with those prescribed other medicines for the same indications/disease	3

**Table 4:** Considerations for study methods and results.

Description of source population (population where the data are obtained)	Name of database, region/country, healthcare system Total number of people in the database, timeframe. Ideally, also the proportion of mother/infant dyads linkable in the database, capture and linkage of outpatient and inpatient prescribing and dispensing	9

Period (dates)	Study period, years included	10

Exposure to the new medicine(s)	Medicine code lists (e.g., ATC, Read, and/or NDC codes) Exposure definition (e.g., reference half-life used to calculate exposure for preconception administration) Exposure duration	9

Exposure to the medicine(s) that could be used as potential comparators	Exposure definition (e.g., reference half-life used to calculate exposure for preconception administration) Exposure duration, coprescription for a similar indication	3

Medicine details	Prescription, reimbursement, dispensing records, medication administration/actual use, duration of treatment, dosage, etc. Formulation Route of administration Comedications	9

Indication for prescription	State how the indication for the medicines studied will be ascertained and why documentation may be incomplete Indicate whether “diagnosis ever” will be applied and time of records Validity of data on indication for prescription. If algorithms are used to establish indication, these should have high specificity and sensitivity, as determined by internal exploration or the available literature	10

Description of pregnancy	Use of birth registers and/or algorithms to identify pregnancies within the database, including multiple pregnancies Best estimates of start and end dates of a pregnancy and how these are obtained. Use of sensitivity analyses where not all pregnancies have ultrasound dating	6

Description of stratifications	Trimesters with definitions Trimester 1: from (1^st^ day) last menstrual period (LMP) to day < 98 after LMP; or end of pregnancy, whichever earlier (i) Trimester 2: from day 98 after (1^st^ day) LMP to day < 196 after LMP; or end of pregnancy, whichever earlier (ii) Trimester 3: from day 196 after (1^st^ day) LMP onwards until end of pregnancy Defining by weeks and days accounts for potential differences in databases	1

Statistical analyses	Descriptive analyses: (i) General characteristics (counts and percentages) (above) (ii) Number of pregnant women in the data source (some may have >1 pregnancy) (iii) Number of pregnancies/births during study period (iv) Number of pregnancies/births that can be linked to infants (v) Number of pregnancies ending in pregnancy loss (vi) Number/proportion of pregnancies exposed to the study medicine (vii) Number/proportion prescribed the medicine more than once (viii) Number/proportion of pregnancies exposed to comparator medicine(s) (ix) Proportion of women of childbearing age with medicated and unmedicated condition under consideration (x) Proportion of women with new medicine and comparator medicines during pregnancy (xi) Proportion of women without documented indication of new medicine and comparator medicines during pregnancy (xii) Proportion of pregnant women prescribed new medicine and comparator medicines (xiii) Proportion of women of childbearing age prescribed new medicine (numerator: users of medicine, denominator: women of childbearing age) Inferential analyses adjusted for covariates listed below. Time-varying covariates may be used, if appropriate Time-trend analyses Interrupted time series, e.g., to consider regulatory interventions Meta-analyses of several databases	11

Methods to address bias	Describe: missing data, potential for misclassification bias, and unmeasured confounding	8

Description of women in the database (this list is not exhaustive)	General descriptive characteristics: (i) Maternal ages (if accurately available) (ii) Maternal socioeconomic status (iii) Marital status, if not colinear with socioeconomic status (iv) Substance misuse, smoking, heavy alcohol use Pregnancy-related conditions (by trimester): (i) Maternal BMI (ii) Gestational diabetes (iii) Gestational hypertension (iv) Preeclampsia (v) Infections and fevers in pregnancy (vi) All medicine used during pregnancy Comorbidities before pregnancy Coprescriptions during pregnancy	5

Length of follow-up in database?	Years of follow-up available	9

No studies reported on data linkage between datasets.

**Table 5:** Discussion.

General and main results	Summary of results, including items based on Table 4, where appropriate	5

Results into context: comparisons with other DUS/sources of information	Findings should be compared with other DUS from other regions/countries or other medicines in same class, or other treatments for the same indication or condition, ideally with putative explanations of any differences and similarities	2

Results into context: relationship to regulatory decisions	Results should be discussed in the context of the regulatory approval status (on- versus off-label use in childbearing or pregnant women) of the newly approved medicine or existing treatment guidelines	1

Limitations	Information on pregnancies Identification of pregnancies: start date might be difficult to identify Information on drug exposure Drug exposure identified by prescriptions (prescribed, dispensed, or reimbursed) in secondary sources may not fully represent actual exposure due to nonadherence or irregular adherence Not all databases have information on indication, duration of prescription Is all exposure captured? For example, are in-hospital administrations, private prescriptions, medicines that are not reimbursed included in the data?	4

Strengths	Nature of the database: Size of datasets Whole population data Representativeness of the national or regional population Ability to capture all types of pregnancies in the database The database best suited to the study depends on the purpose of the study. Explain selection of database Timing of data collection: prospectively collected data is free of recall bias	3

Generalisability	Completeness of data source; number/extent of geographies covered Any regional differences may be related to prescribing guidelines or custom and practice Representativeness of overall studied population (e.g., not only the better educated, nonsmokers, and older mothers)	3

Conclusion	Is the medicine being used by childbearing/pregnant women? Is the medicine prescribed in primary, secondary, or tertiary care? When in pregnancy, for how long in pregnancy, what doses, for what indications, time trends?	2

## Data Availability

All data is presented in the manuscript and supplement.

## Appendix

### Search Terms Used to Identify Drug Utilization Studies

The following keywords were used in different databases to identify all drug utilization studies:

PubMed: (((((“Cohort Studies”[Mesh] OR Registries[Mesh] OR cohort[tiab] OR follow-up[tiab] OR prospective[tiab] OR observational[tiab] OR longitudinal[tiab] OR retrospective[tiab] OR birth registr^∗^[tiab] OR population-based[tiab] OR population health data[tiab]) AND (prevalence[mesh] OR prevalen^∗^[Title/Abstract] OR change^∗^[Title/Abstract] OR trend^∗^[Title/Abstract] OR pattern^∗^[Title/Abstract] OR adhere^∗^[Title/Abstract] OR trajector^∗^[Title/Abstract] OR continu^∗^[Title/Abstract] OR discontinu^∗^[Title/Abstract]) AND (pharmaceutical preparations[Mesh] OR drug utilization[mesh] OR drug^∗^[Title] OR medicat^∗^[Title] OR prescript^∗^[Title/abstract] OR prescrib^∗^[Title/abstract] OR medicin^∗^[Title] OR dispens^∗^[Title/abstract] OR pharmacoepidemiology[Mesh] OR drug therapy[Mesh]) AND (pregnancy[mesh] OR pregnancy trimesters[mesh] OR pregnan^∗^[Title] OR matern^∗^[Title] OR gestation^∗^[Title] OR perinatal^∗^[Title] OR peri-natal^∗^[Title] OR prenatal^∗^[Title] OR antenatal^∗^[Title] OR ante-natal^∗^[Title] OR gravid^∗^[Title]) NOT (“animals”[MeSH] NOT “humans”[MeSH])) AND (english[Filter]))) AND ((“2015/01/01”[Date - Publication]: “3000”[Date - Publication]))

Embase: (“cohort analysis”/mj OR “follow up”:ti,ab,kw OR cohort:ti,ab,kw OR prospective:ti,ab,kw OR observational:ti,ab,kw OR longitudinal:ti,ab,kw OR retrospective:ti,ab,kw OR “birth registr^∗^”:ti,ab,kw OR “population based”:ti,ab,kw OR “population health data”:ti,ab,kw OR “register”/mj) AND (“drug”/mj OR “drug utilization”/mj OR “pharmacoepidemiology”/mj OR “drug therapy”/mj OR drug^∗^:ti OR medicat^∗^:ti OR prescript^∗^:ti,ab,kw OR prescrib^∗^:ti,ab,kw OR medicin^∗^:ti OR dispens^∗^:ti) AND (“pregnancy”/mj OR pregnan^∗^:ti OR matern^∗^:ti OR gestation^∗^:ti OR perinatal^∗^ OR “peri natal”:ti OR prenatal^∗^:ti OR antenatal^∗^:ti OR “ante natal^∗^”:ti OR gravid^∗^:ti) AND (“prevalence”/mj OR prevalen^∗^:ti,ab,kw OR change^∗^:ti,ab,kw OR trend^∗^:ti,ab,kw OR pattern^∗^:ti,ab,kw OR adhere^∗^:ti,ab,kw OR trajector^∗^:ti,ab,kw OR continu^∗^:ti,ab,kw OR discontinu^∗^:ti,ab,kw) NOT (“animal”/exp NOT “human”/exp) AND [2015-2020]/py AND [english]/lim excluding medline

EU PAS Register: “observational study” (in study type), “pregnant women” (in population), and “drug”, “drug' “drug utilisation study” (in scope of the study)

## Abbreviations

ADRs: Adverse drug reactions
DUS: Drug utilization studies
IMI: Innovative Medicines Initiative
UK: United Kingdom.

## Additional Points

*Note on Terminology*. In the term “drug utilisation study” (DUS), “drug” is used adjectivally, in line with MeSH terminology. When referring to medicinal products, we have used the term “medicine,” as this paper only describes prescribed products used in the treatment of illness (OED medicine, n.1: *Oxford English Dictionary* (http://oed.com)). There are no references to recreational drugs in this paper. See the following: Glossary breastfeeding confounding WP1 Document Template.docx (http://live.com). *Five Key Points*. (i) When medicines are introduced to the market, there is limited information available related to human pregnancy, placental transfer, foetal elimination, childbirth, lactation, and breastfeeding. This study lists and summarises key information items to be reported in a drug utilization studies for new medicines that may be used in pregnancy. (ii) A DUS can confirm at an early stage whether there are sufficient exposed pregnancies in available data sources to ensure a safety study is powered to detect a difference in the prevalence of adverse pregnancy or infant outcomes or if additional data from other databases are needed. (iii) A DUS may also help address methodological considerations, such as selection of comparators. (iv) A DUS to understand pregnancy exposure may be embedded in a DUS in the general population, in a cohort of women of childbearing age, or in a cohort of pregnant women. (v) In-hospital prescribing is an important consideration. *Plain Language Summary (PLS)*. DUS were defined by the World Health Organization as descriptive or analytical reports of the marketing, distribution, prescription, and use of medicinal products in a society, with emphasis on the resulting medical and socioeconomic consequences [10]. Studies focusing on safety outcomes typically require large populations to characterise the patient groups exposed to the medicines under investigation. However, there is often less information for subpopulations, such as pregnant or breastfeeding women, particularly when new medicines are considered. It is important to understand what information can be obtained from drug utilization studies (DUS) involving pregnant women, when the drug is new to the market. The objective is to identify and review DUS for new medicines in pregnancy and breastfeeding and to list and summarise key information items to be reported. The 11 identified publications reported the medicine's use for the first 3 to 5 years after marketing approval. No reports assessed utilization in the first 3 years of approval. It was usual to issue interim reports annually. A DUS can confirm at an early stage whether there are sufficient exposed pregnancies in available data sources to ensure a safety study. A DUS may also help address methodological considerations such as selection of comparators. This review summarises key aspects of a DUS for new medicines in pregnancy. Such data improve the interpretation and analyses of safety studies.
